# Integrated phenotypic-genotypic approach to understand the influence of ultrasound on metabolic response of *Lactobacillus sakei*

**DOI:** 10.1371/journal.pone.0191053

**Published:** 2018-01-25

**Authors:** K. Shikha Ojha, Catherine M. Burgess, Geraldine Duffy, Joseph P. Kerry, Brijesh K. Tiwari

**Affiliations:** 1 Food Chemistry and Technology, Teagasc Food Research Centre, Dublin, Ireland; 2 Food Safety, Teagasc Food Research Centre, Dublin, Ireland; 3 Food Packaging Group, University College Cork, Cork, Ireland; University of Torino, ITALY

## Abstract

The lethal effects of soundwaves on a range of microorganisms have been known for almost a century whereas, the use of ultrasound to promote or control their activity is much more recent. Moreover, the fundamental molecular mechanism influencing the behaviour of microorganisms subjected to ultrasonic waves is not well established. In this study, we investigated the influence of ultrasonic frequencies of 20, 45, 130 and 950 kHz on growth kinetics of *Lactobacillus sakei*. A significant increase in the growth rate of *L*. *sakei* was observed following ultrasound treatment at 20 kHz despite the treatment yielding a significant reduction of ca. 3 log cfu/mL in cells count. Scanning electron microscopy showed that ultrasound caused significant changes on the cell surface of *L*. *sakei* culture with the formation of pores “sonoporation”. Phenotypic microarrays showed that all ultrasound treated *L*. *sakei* after exposure to various carbon, nitrogen, phosphorus and sulphur sources had significant variations in nutrient utilisation. Integration of this phenotypic data with the genome of *L*. *sakei* revealed that various metabolic pathways were being influenced by the ultrasound treatments. Results presented in this study showed that the physiological response of *L*. *sakei* in response to US is frequency dependent and that it can influence metabolic pathways. Hence, ultrasound treatments can be employed to modulate microbial activity for specialised applications.

## Introduction

A range of agri-food and bio-processing applications offered by ultrasound has been widely documented. In the past two decades, ultrasound has gained renewed interest for modifying the cell membrane permeability, thus delivering gene or macromolecules, chemotherapeutic drug and genetic materials into cell nuclei. However, the efficiency at which these molecules were delivered into the cell varies considerably between studies owing to various extrinsic and intrinsic control parameters [1]. Ultrasound induced cell permeabilisation has been termed as “sonoporation”, which can be defined as the transient and reversible increase in the permeability of plasma cell membranes when exposed to ultrasonic waves [2]. Sonoporation results in the formation of pores on microbial cell membranes, thereby providing a channel for transport of essential nutrients and removal of toxic substances across these membranes. Such an alteration of the membrane bilayer has been reported to affect cellular functions such as nutrient transport, enzymes activities, and cell growth and multiplication. Considering this, appropriate selections of ultrasound intensities are crucial for the viability of cells and enhancing the production of bioactive metabolites for specialised applications [3, 4]. However, an increase in ultrasonic power or exposure time can lead to inactivation or cell death due to thinning/alteration of cell membranes resulting in leakage of cellular content, localized heating, and production of free radicals. The effectiveness of ultrasound on microorganisms is strongly influenced by various factors, including but not limited to, microbial ecology (e.g. microorganism type, growth medium and composition), ultrasound parameters (e.g. ultrasonic power, intensity and frequency), exposure time, pH and temperature [5]. For example, studies have shown that Gram-positive bacteria are more resistant to ultrasound compared to Gram-negative bacteria, possibly because Gram-positive bacterial cells possess a thick and more robust cell wall due to cross-linking of peptidoglycan and teichoic acid [6].

Ultrasound can enhance the growth rate of microbial cells due to its ability to increase the rate of transport of oxygen and nutrients to the cells and increase the rate of transport of waste products away from the cells. Studies have shown the effect of ultrasound on viability, physiological characteristics and growth at various stages. For example Lanchun, Bochu [7],[8] reported that the appropriate ultrasonic frequency can accelerate the growth of *Saccharomyces cerevisiae* by nearly 33.3%, with the stationary phase reached four hour earlier compared to the control. Similarly, Jomdecha and Prateepasen [9] studied the effect of pulse ultrasonic irradiation on the lag phase of *S*. *cerevisiae*. The lag durations were changed significantly due to ultrasonic energies and durations. In particular, application of sufficient amounts of ultrasonic energies were shown to reduce the lag time, resulting in accelerated yeast growth whereas, a higher level of ultrasonic energy delayed growth by increasing the lag phase. High intensity ultrasound treatment of milk containing *Bifidobacterium* sp. has been shown to enhance the production rates of organic acids and the growth of *Bifidobacterium* sp., while concurrently reducing fermentation time [10, 11]. Similarly, Yang, Zhang [12] demonstrated that ultrasound pre-treatment, followed by incubation, enhances the growth of *Brevibacterium* sp.

Lactic acid bacteria (LAB) are Gram-positive microorganisms which are widespread in nature and adapted to grow under different environmental conditions. Application of *Lactobacillus sp*. in food products have shown to inhibit the growth of pathogenic and spoilage microorganisms and improve organoleptic properties of food [13, 14]. Additionally, *Lactobacillus* sp. are reported to have beneficial effects on gut health and play a vital role in the alleviation of metabolic diseases [15, 16]. *Lactobacillus sakei* is a lactic acid bacterium of commercial importance because of its ability to ferment various nutrients available in food. *L*. *sakei* is regarded as a safe strain of LAB, with an ability to grow at diverse environmental conditions, including low temperatures and high salt concentrations.

Genomics and/or proteomics approaches are being used to investigate the microbial response to environmental conditions, which provides valuable information at the point of cell harvesting. A high-throughput assessment of metabolic activities of cells can be achieved by employing a phenotype microarray (PM) technique. The PM technique has highlighted differences in growth requirements, nutrient utilisation, sensitivity to toxins, and genetic diversity in bacteria, fungi and mammalian cells. The PM technique is a convenient way of measuring live cell performance under different environmental conditions [17]. Hence this technique was employed in this study to understand the metabolic changes occurring at phenotypic level. The objective of this study was to investigate the effect of different ultrasound frequencies on growth, morphology, viability and metabolic activities of *L*. *sakei*.

## Methods

### Culture and sample preparation

*L*. *sakei* DSM 15831 was obtained from DSM, Germany. The bacteria were cultured in de Man Rogosa Sharpe (MRS) broth (Oxoid Ltd, Cambridge, UK) medium at 30°C in a microaerophilic chamber for 48 h. Microbial cells were harvested at 7000×g for 10 min and subsequently added to either to MRS broth or phosphate buffer saline (PBS) to achieve a target population of ca. 10^7^ cfu/mL for subsequent ultrasound treatment and analysed further. MRS broth and PBS (Oxoid Ltd, Cambridge, UK) were prepared as per the manufacturer’s instruction.

### Ultrasound treatment

Different ultrasound frequency exposures were examined using a 20 kHz probe system and multiple bath systems operating at frequencies of 45, 130 and 950 kHz. For the 20 kHz ultrasound probe system, a *L*. *sakei suspension* (100 mL) was placed in a sterile container (250 mL) and sonicated (for 10 min) by submerging the probe tip (Ø 1.9 cm) (Model: 500 HD, Hielscher Ultrasonics GmbH, Germany) into the bacterial suspension. In the case of the bath systems, treatments were carried out by placing the sterile flat bottom glass tubes (30 mL) containing 20 mL of *L*. *sakei* suspension into the ultrasonic baths of 45 kHz (Elma Schmidbauer GmbH, Germany), 130 kHz (Elma Schmidbauer GmbH, Germany) and 950 kHz (Kaijo Shibuya America Inc.) for 1 h. Samples without ultrasound treatment were considered as the control. These conditions were selected based on prior studies carried out to establish exposure time. For all treatments, the temperature was maintained at 4.0±1.0°C by circulating cold water using a temperature-controlled refrigerated water circulation system. Three biological replicates were carried out for each of the treatment. The energy input (power, W) was measured using calorimetric method outlined previously by Tiwari, Muthukumarappan [18]. Acoustic cavitation pressures namely direct field pressure (Po, kPa), stable cavitation pressure (Ps, kPa) and transient cavitation pressure (Pt, kPa) were measured using a Hydrophone (HCT-3010) attached to a cavitation meter (Model: MCT-2000, Onda Co). Various ultrasonic parameters obtained are listed in Table 1.

**Table 1:** Ultrasound parameters for various frequencies investigated.

**Ultrasound frequency (kHz)**	**20.0**	**35.0**	**45.0**	**130.0**	**950.0**
**Direct field pressure (Po, kPa)**	3.0	42.0	40.0	26.0	42.0
**Stable cavitation pressure (Ps, kPa)**	4.0	19.0	18.0	13.0	6.0
**Transient cavitation pressure (Pt, kPa)**	23.0	25.0	21.0	9.0	3.0
**Ultrasonic power (W)**	155.3	6.9	5.5	7.2	2.7

### *L*. *sakei* growth measurement

The growth of control and ultrasound treated *L*. *sakei* was monitored by measuring a change in optical density (OD) at a regular interval of 30 min intervals using 96 well plates in a temperature controlled microbial growth analyser (Multiskan^TM^ Microplate Spectrophotometer, Thermo Scientific) at a wavelength of 595 nm and a temperature of 30°C over a 24 h incubation time. Population of control and ultrasound treated *L*. *sakei* cultures (immediately after treatment) were enumerated in duplicate on MRS pour-plates after 72 h incubation at 30°C.

### Phenotypic microarray assay

The control and ultrasonic treated *L*. *sakei* were examined for phenotypic divergence using an Omnilog™ phenotypic microarrays (using PM 1 to 4) (BioLog Inc., Hayward, California). Control and ultrasound treated *L*. *sakei cell* suspensions were prepared and PM plates were inoculated by following manufacturers’ instructions. Control and ultrasound treated cell suspensions (ca. 10^5^ cells/ml) were prepared and 50 μL of the cell suspension were inoculated into the wells of the PM panels. These plates were incubated at 30°C for 48 h in an Omnilog™ micro plate reader (BioLog Inc., Hayward, California). The digital imagery of this instrument tracks changes in the respiration of *L*. *sakei cultures* growing in individual wells over the incubation time. Incubation and recording of phenotypic data was performed automatically by the OmniLog^TM^ instrument. The Omnilog™ output for a given plate consists of an OD reading for each well, recorded at 15 min intervals over the 48 h incubation period. The data output for the control and ultrasound treated *L*. *sakei* cells were analysed using DuctApe software [19]. Negative controls (wells containing the inoculated Omnilog™ growth medium, but without any substrate for each PM plate was used to normalise differences in inoculums and redox dye oxidation between samples) were subtracted from each reading for each plate.

### Genotypic and phenotypic analysis

DuctApe was employed for the analysis of genomic and phenomic data to obtain metabolic differences occurring due to ultrasound treatment. Briefly, three modules of DuctApe software suite include 1) dgenome for genomic data analysis of *L*. *sakei* subsp. *carnosus* DSM 15831 (Control); 2) dphenome for PM data analysis (PM 1–4) of the ultrasound treated and control; 3) dape for combined analysis of genomics and phenomics for metabolic reconstruction according to the Kyoto Encyclopedia of Genes and Genomes (KEGG) database [20], by combining outputs of the dgenome and dphenome modules. The genome sequence of *L*. *sakei* subsp. *carnosus* DSM 15831 was obtained from National Center for Biotechnology Information (NCBI)[21]. NCBI database resource organises information on various characterstics of range of organisms including genomic sequences. The genome of *L*. *sakei* subsp. *carnosus* DSM 15831 has a symmetrical identity of 88.005% with *L*. *sakei* subsp. *sakei* 23K. *L*. *sakei* subsp. *carnosus* used in this study is a type strain CCUG 31331 (R 14 b/a, DSM 15831) which has shown genomic variation within the subspecies *carnosus* [22]. Intraspecies genomic diversity among *L*. *sakei* population has been extensively studied [23]. Strain diversity among *L*. *sakei* populations and its linkages with evolutionary histories studied by Chaillou, Lucquin [24] has shown variations among genomic sequences and protein patterns. The protein pattern of *L*. *sakei* subsp. *carnosus* has been reported to be similar to that of the *L*. *curvatus* subsp. *Melibiosus*, with a significantly different pattern obtained from *L*. *sakei* subsp *sakei* [25].

Experimental phenotypic microarray datasets obtained from the OmniLog™ Phenotype Microarray (PM) technology for the ultrasound treated and control samples were analysed with the DuctApe software (Ver 0.16.2) suite capable of combining genotypic and phenotypic data[19]. DuctApe programme code written in Python was carried out on Ubuntu Version 15.05.

### Imaging

Cryo−scanning electron microscopy (SEM) was carried out using the Zeiss Ultra Plus Field Emission Scanning Electron Microscopy with a Quorum Cryo Preparation chamber. Samples were plunged into liquid Nitrogen slush (-190°C) and transferred, under vacuum, into a sublimation/fracture/coating chamber. Here the samples were freeze fractured to expose the internal structure, sublimated at -100°C for 20 min and coated in platinum. The samples were held under vacuum and transferred onto a cold stage in the SEM where it was maintained at a temperature of -170°C and imaged.

### Mathematical modelling and statistical analysis

To evaluate the sigmoidal shape of the *L*. *sakei* growth curve, the primary growth parameters were obtained by fitting OD_595nm_ versus incubation time to three models namely Scale free, Biphasic and the modified Gompertz model using the DMFit excel based tool [26]. Readers are referred to Baranyi and Roberts [26] and Baranyi, Roberts [27] for theoretical derivation and to obtain information about the explicit form of the models employed to obtain the primary growth parameters. The primary growth kinetic parameters of *L*. *sakei* populations following US treatment i.e. specific growth rate (μ_max_, OD unit/h) and lag phase (λ, h) were calculated. It must be noted that the μ_max_ obtained by fitting these models is only a potential rate because, theoretically this exact rate cannot be obtained due to the applied limiting functions which has effects even in the linear phase of the growth curve. However, the difference between real rate and rate obtained by the model is negligible if the m_Curve_ curvature parameter obtained from the model fit is sufficiently large [26].

Goodness of model fit was analysed based on coefficient of regression (R^2^), Root Mean Squared Error (RMSE) and by analysing residuals, experimental vs predicted OD values. Analysis of Variance (ANOVA) and Tukey’s test was carried out to separate means using SAS statistical software (SAS Ver 9.3). Mean values were considered significant at P<0.05. PROC CORR procedure of SAS was carried out to obtained correlation between various parameters.

## Results

### Effect of ultrasound on *L*. *sakei* population and growth curve

**Fig 1:**
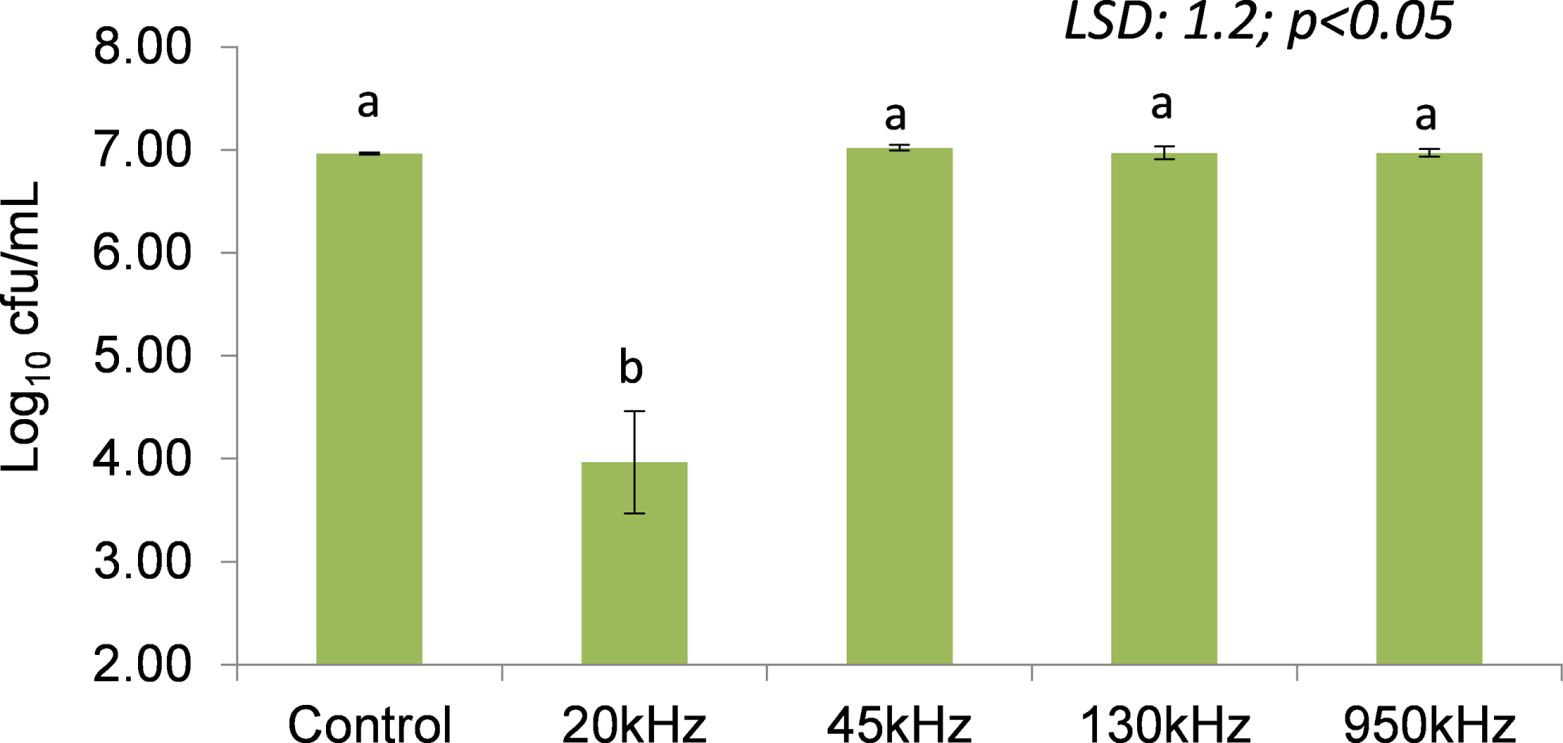
*L*. *sakei* population of control (untreated) and surviving population after treatment at various ultrasound frequencies of 20, 45, 130 and 950 kHz. (^ab^Columns with similar letters are significantly different at P<0.05; LSD: Least significant difference).

Fig 1 shows the effect of ultrasound on the inoculated *L*. *sakei* population (6.96 log cfu/mL) using a plate count method immediately after treatment at various US frequencies. The surviving *L*. *sakei* population ranged from 3.96 log cfu/ml (20 kHz) to 7.02 log cfu/mL (45 kHz). No significant changes (P<0.05) in *L*. *sakei* population were observed with respect to ultrasound frequency and control, with an exception of 20 kHz, where a significant (P<0.05) decrease of 3 log cfu/mL was observed.

**Fig 2:**
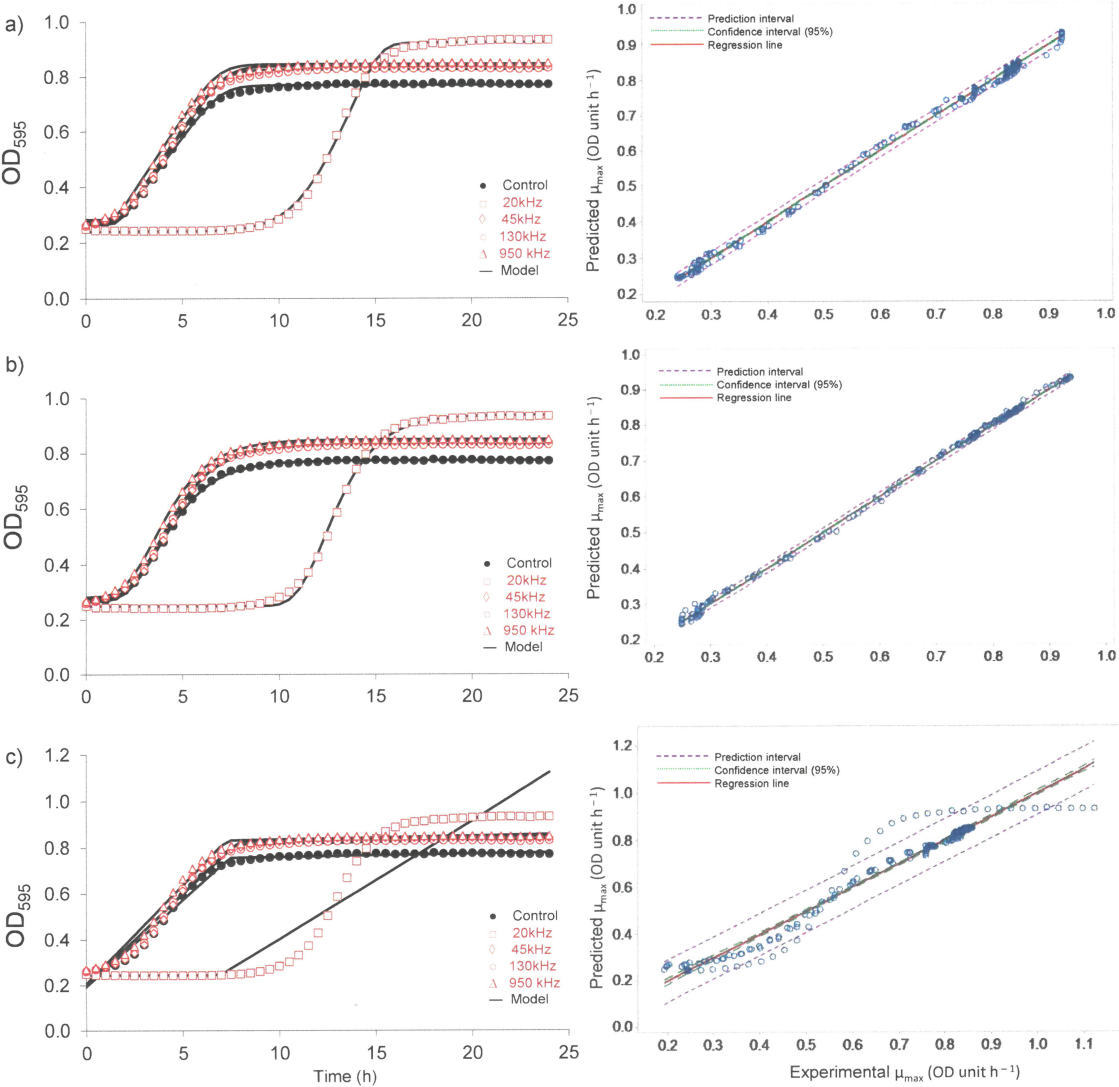
*L*. *sakei* growth curve plot as measured by optical density over a 25 h incubation at 30°C following various ultrasound treatments plotted using Scale free (a), Gompertz (b) and Biphasic models (c) and corresponding model fitting.

The growth of the surviving *L*. *sakei* cells was monitored over a 24 h period using optical density measurements at 595nm. Fig 2 shows the plotted average optical density at each incubation time (h) for *L*. *sakei* subjected to ultrasound treatments at various frequencies of 20 kHz, 45 kHz, 130 kHz, 950 kHz and control (no ultrasound treatment). The initial OD_595nm_ was in a range of 0.243 to 0.284 OD units irrespective of the treatment.

As can be seen from Fig 1 the starting population of *L*. *sakei* following US treatment was in the range of 3.96 log cfu/mL (20 kHz) to 6.96–7.04 cfu/mL for all other treatments and control. The growth rate of the control remained lower at all times compared to ultrasound treated samples, irrespective of frequencies employed. It should be noted that different systems were employed for the ultrasound treatment which operated at different frequencies. In the case of the 20 kHz treatment, *L*. *sakei* showed a longer lag time followed by a subsequent high growth rate. As can be seen from Fig 2, the slope of the exponential phase of treated and control *L*. *sakei* cells were parallel, indicating the growth behaviour was relatively similar. However, a significant increase in lag time was observed for samples treated with 20 kHz frequency.

Table 2 shows the growth parameters obtained by fitting OD_595nm_ versus incubation time (h) to three models, namely scale free, the Gompertz and biphasic model. Three models were employed to obtain key growth parameters of specific growth rate (μ_max_, OD unit/h) and lag phase (λ, h). μ_max_ and λ were considered as key parameters required to investigate the effect of treatment on the growth behaviour of *L*. *sakei*. All three models gave the highest μ_max_ (OD unit/h) and λ (h) for *L*. *sakei* samples treated with 20 kHz and lowest for control. Initial (Y_0_) and final (Y_max_) absorbance ranged from 0.251 to 0.273 and 0.813 to 0.947 respectively. In general, all three models investigated to describe the growth behaviour of *L*. *sakei* predicted well with an exception for *L*. *sakei* subjected to 20 kHz. The biphasic model failed to predict growth behaviour for 20 kHz treated *L*. *sakei* samples (Fig 2C). μ_max_ obtained by three models ranged from 0.099–0.122 OD unit/h, 3.85×10^−10^–0.084 OD unit/h and 0.122–0.166 OD unit/h whereas, λ ranged from 1.56–11.51 h, 7.33–9.39 h and 1.95–12.536 h for scale free, biphasic and the Gompertz model respectively. Predicted growth models tested for the goodness of model fit by using R^2^ and RMSE values, showed that the R^2^ values for all models were >0.902 and RMSE values were <0.017 for all growth curves. However, the biphasic model showed higher RMSE values (0.017–0.095) and low R^2^ values (0.90–0.99) compared to the scale free and the Gompertz model across all treatments including the control. A plot between model predicted and experimental OD values showed that values for both the Gompertz and scale free models were within the prediction and 95% confidence interval whereas, values obtained from the biphasic model did not show good model prediction (Fig 2). Further, the Biphasic model predicted low values for growth parameters compared to the other two models. Growth model parameters provided by both the Gompertz and scale free models were comparable. Considering goodness of fit, model predicted values and residual analysis, the Gompertz model was found to be best suited to describe the growth model for all treatments. The Gompertz model showed a good fit with the experimental data with low standard error (SE) of < 0.042 and high regression coefficients (*R*^*2*^) of > 0.985. Analysis of growth curve for control and ultrasound treated *L*. *sakei samples* using Gompertz will be discussed here after.

**Table 2:** *L*. *sakei* growth parameters along with 95% confidence interval, regression coefficient (R^2^), root mean square error (RMSE) of model fit obtained for control and ultrasound treated *L*. *sakei* cells.

Treatment	Model	μ_max_ (OD unit h− ^1^)	Lag time (λ, h)	Y_0_ (OD unit)	Y_max_ (OD unit)	R^2^	RMSE
**Control**	**Scale free**	0.099±0.003 ^a^ (0.094–0.104)	1.707±0.034 ^b^ (1.640–1.773)	0.259	0.813	0.998	0.008
**20 kHz**	** **	0.122±0.057 ^a^ (0.011–0.233)	11.517±0.494 ^a^ (10.549–12.486)	0.251	0.947	0.999	0.011
**45 kHz**	** **	0.103±0.001 ^a^ (0.102–0.105)	1.790±0.025 ^b^ (1.741–1.838)	0.270	0.840	0.997	0.009
**130 kHz**	** **	0.105±0.001 ^a^ (0.103–0.107)	1.925±0.024 ^b^ (1.877–1.972)	0.269	0.849	0.998	0.009
**950 kHz**	** **	0.104±0.001 ^a^ (0.101–0.106)	1.560±0.022 ^b^ (1.517–1.603)	0.273	0.850	0.997	0.010
	***LSD***	0.0441	0.3781				
**Control**	**Gompertz**	0.122±0.003 ^c^ (0.116–0.129)	2.113±0.039 ^b^ (2.037–2.188)	0.259	0.813	0.999	0.005
**20 kHz**	** **	0.166±0.001 ^a^ (0.164–0.168)	12.536±1.888 ^a^ (8.836–16.236)	0.251	0.947	0.999	0.010
**45 kHz**	** **	0.128±0.001 ^b^ (0.126–0.129)	2.187±0.022 ^b^ (2.145–2.230)	0.270	0.840	0.999	0.005
**130 kHz**	** **	0.129±0.001 ^b^ (0.127–0.132)	2.326±0.023 ^b^ (2.281–2.370)	0.269	0.849	0.999	0.005
**950 kHz**	** **	0.128±0.002 ^b^ (0.124–0.132)	1.955±0.026 ^b^ (1.904–2.006)	0.273	0.850	0.999	0.004
	***LSD***	0.0033	1.4502				
**Control**	**Biphasic**	0.079±0.002^b^ (0.075–0.082)	7.617±0.124 ^a^ (7.373–7.861)	0.259	0.813	0.989	0.017
**20 kHz**	** **	3.85±1.12×10^−10^ ^c^ (1.64–6.06×10^−10^)	9.396±2.734 ^a^ (4.038–14.754)	0.251	0.947	0.902	0.095
**45 kHz**	** **	0.080±0.001 ^b^ (0.078–0.082)	7.710±0.032 ^a^ (7.647–7.773)	0.270	0.840	0.988	0.020
**130 kHz**	** **	0.079±0.001 ^b^ (0.078–0.080)	7.966±0.082 ^a^ (7.804–8.127)	0.269	0.849	0.986	0.022
**950 kHz**	** **	0.084±0.001 ^a^ (0.082–0.087)	7.334±0.049 ^a^ (7.238–7.430)	0.273	0.850	0.990	0.019
	***LSD***	0.0019	2.0728				

(^abc^Values with different letters are significantly different at P<0.05; LSD: Least significant difference)

Correlation analysis (Table 3) shows that the stable cavitation pressure (r = 0.643; P<0.01) and standing wave pressure (r = 0.489; P<0.05) had a positive effect on μ_max_ (OD unit/h) whereas, both the stable (r = 0.580; P<0.05) and transient (r = 0.509; P<0.05) cavitation pressure showed positive correlation. The *L*. *sakei* population was significantly affected by ultrasonic power as evident by high correlation coefficient between power and log (cfu/mL) (r = -0.977; P<0.0001) and hence, it can be concluded that the higher power can inactivate bacterial cells. Surprisingly, low microbial count observed for 20 kHz samples showed longer lag phase followed by significantly higher growth rate.

**Table 3:** Correlation analysis showing relationship between various ultrasonic parameters and *L*. *sakei* growth parameters.

	**Fo** **(kPa)**	**Po** **(kPa)**	**Ps** **(kPa)**	**Pt** **(kPa)**	**Power (W)**	**μ**_**max**_ **(OD unit/h)**	**λ** **(h)**
**Fo (kPa)**	1.000						
**Po (kPa)**	0.389^ns^	1.000					
**Ps (kPa)**	-0.476*	0.619**	1.000				
**Pt (kPa)**	-0.827****	-0.246^ns^	0.410 ^ns^	1.000			
**Power (W)**	-0.324 ^ns^	-0.923****	-0.640**	0.408 ^ns^	1.000		
**μ_max_ (OD unit/h)**	-0.228 ^ns^	0.489*	0.643**	0.420 ^ns^	-0.376 ^ns^	1.000	
**λ (h)**	-0.297 ^ns^	0.368 ^ns^	0.580*	0.509*	-0.238 ^ns^	0.976****	1.000
**Log (cfu/mL)**	0.273 ^ns^	-0.367^ns^	-0.557**	0.229^ns^	-0.977****	-0.998****	1.000

^ns^Not significant (P<0.05)

*significant at P<0.05

**significant at P<0.01

****significant at P<0.0001.

The specific growth rate (μ_max_, OD units/h) and lag phase (λ, h) obtained from Gompertz model for the control sample was 0.122±0.003 OD units/h (CI: 0.116–0.129) and 2.113±0.039 h (CI: 2.037–2.188) respectively. Significantly (P<0.05) higher μ_max_ was observed for 20 kHz (0.166±0.001 OD units/h) compared to other frequencies investigated and the control. In general ultrasound treated samples showed significantly higher μ_max_ compared to the control. No significant differences were observed for μ_max_ with respect to 45, 130 and 950 kHz even though μ_max_ was significantly higher compared to the control. In the case of λ, no significant differences (P<0.05) were observed for control and ultrasound frequencies of 45, 130 and 950 kHz. Significantly higher λ (12.536±1.888 h (CI: 8.836–16.236 h)) was observed for *L*. *sakei* samples subjected to 20 kHz frequency compared to the control and other frequencies investigated.

### Genomic and phenomic characteristics

The genomic sequence of *L*. *sakei* subsp. *carnosus* DSM 15831 was used to study the metabolic response of *L*. *sakei* following ultrasound treatment. The control *L*. *sakei* strain showed proteome/genome size of 1904, out of which 1007 were mapped to KEGG with 877 KEGG orthology IDs, 95 pathways, 829 reactions and 586 unique reactions based on genomic sequence available for the *L*. *sakei* strain employed (S1 Fig). As can be seen only 53.4% of genes were mapped to KEGG because most of genes are not involved in metabolism hence were not annotated through KEGG.

The metabolic abilities of control and ultrasound treated *L*. *sakei* were tested using the PM system (Biolog). Using PM plates 1 to 4, 571 different growth conditions were tested, including 190 different carbon sources, 95 nitrogen sources, 59 phosphorus sources and 35 sulphur sources. Metabolically active conditions were scored using a threshold calculation based on growth curve data. The activity index (AV) was calculated using five clusters (k = 5) which were chosen through an elbow test.

**Fig 3:**
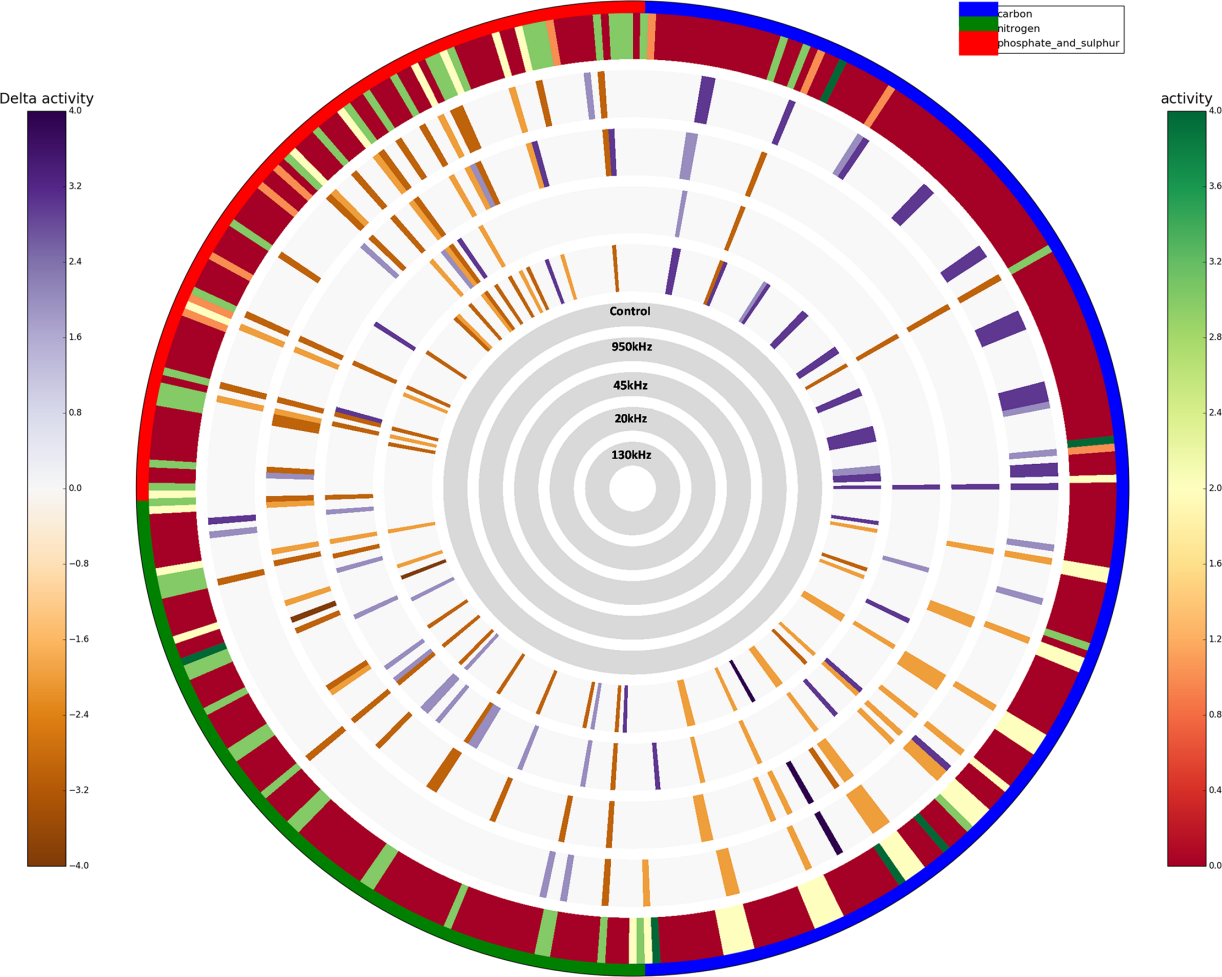
High-throughput metabolic activity in difference mode: The difference with the AV value of control culture is reported when AV≥2; it is grey otherwise; purple indicates a higher activity to; orange colour indicates a lower activity of ultrasound treated samples with respect control.

Unique metabolic functions represent the number of wells for which a single treatment shows an exclusive phenotype, with respect to the others. Results showed that the highest number of unique “more active” metabolic features was detected in PM plates inoculated with 20 kHz treated culture and control (N = 31 and N = 23 respectively) suggesting a higher metabolic potential of these strains as compared to the others. Whereas, the 20 kHz treated culture and control culture strains exhibited “less active” phenotypic traits in only one and five conditions respectively, as shown in Table 4. In contrast, the 45 kHz treated culture seems to be the least metabolically active strain, as it showed a lower number of “more active” metabolisms (N = 9) and the highest number of “less active” metabolisms (N = 19). When looking at the single PM compound categories, the control culture was able to metabolise 19.27% of carbon sources, 25.00% of nitrogen sources and 31.25% of phosphorus and sulphur sources (Table 4). The *L*. *sakei* culture treated at 950 kHz frequency showed highest metabolic activity (24.48%) in carbon plates followed by 130 kHz (22.91%), 20 kHz (19.27%), Control (19.27%) and 45 kHz (7.81%). In the case of nitrogen plate, culture treated at 20 kHz had highest activity (31.25%) followed by control (25.00%), 950 kHz (21.87%), 130 (18.75%) and 45 kHz (8.33%). The control showed highest activity (31.25%) for phosphorus and sulphur sources followed by 20 kHz (26.04%), 45 kHz (18.75%), 130 kHz (14.58%) and 950 kHz (13.54%). Here, all the phenotypes were grouped based on the type of PM source molecules. Individual differences among each of the 571 PM metabolic conditions of the cultures treated at different US conditions were then assessed and represented in detail as circular plots (Activity Ring). Any difference in higher/lower metabolic reaction between the reference control culture and the culture treated at different ultrasonic conditions can be visualised using the ‘diff mode’ (Fig 3). Fig 3 shows concentric circles (from outer to inner ring) represent control *L*. *sakei* and delta activity of *L*. *sakei* treated at 950, 45, 20 and 130 kHz respectively, whereas each radial strip corresponds to a single tested phenotype. The higher colour intensity appeared in the carbon plates in the case of 950 and 130 kHz, confirming the lower metabolic activity of the control as compared to the culture treated at 950 and 130 kHz.

**Table 4:** Unique and metabolic activity statistics obtained from DuctApe analysis.

**Unique metabolic functions (% of wells with ∆ ≥ 1)**					
	Control	20 kHz	45 kHz	130 kHz	950 kHz
**More active**	23	31	9	8	5
**Less active**	5	1	19	3	6
**Percentage (%)**	7.29	8.33	7.29	2.86	2.86
**Metabolic activity statistics (% of active wells with AV ≥2)**					
**Carbon sources**	19.27	19.27	7.81	22.91	24.48
**Nitrogen sources**	25.00	31.25	8.33	18.75	21.87
**Phosphorus and sulphur sources**	31.25	26.04	18.75	14.58	13.54

**Fig 4:**
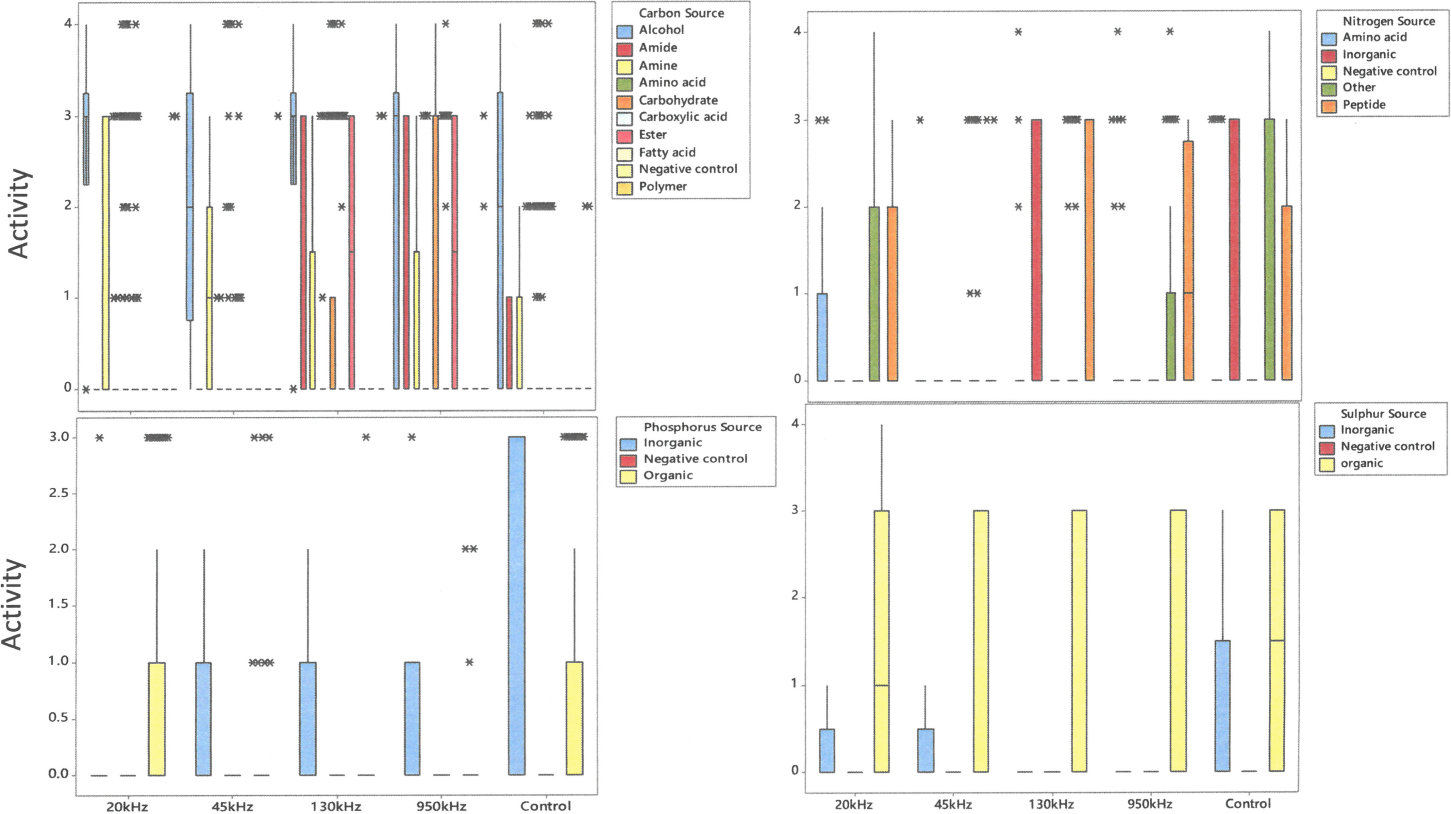
The utilisation of various carbon, nitrogen, phosphorus and sulphur sources by *L*. *sakei* subjected to ultrasonic frequencies of 20 kHz, 45 kHz, 130 kHz, 950 kHz and control, (*indicates outliners).

The utilisation of carbon (C), nitrogen (N), phosphorus (P) and sulphur (S) by the *L*. *sakei* strain subjected to ultrasound treatment is shown in Fig 4. As can be seen from Fig 4, the utilisation of nutrients was significantly affected due to ultrasound treatment. Significant variations in the utilisation of carbon sources was observed for ultrasound treated and control samples. The control was able to ferment various sugars including ribose, lyxose, glucosamine, 2 deoxy-D ribose, arabinose and tagatose whereas low utilisation (AV<2) was observed for melibiose. Ultrasound treated *L*. *sakei* at 130 kHz and 950 kHz showed higher D-mannose, dulcitol, thymidine, sucrose, uridine, 2`-deoxyadenosine, adenosine, D-cellobiose and inosine as a carbon source compared to control or 20 kHz. In the case of carboxylic acids, relatively higher activities for butyric acid, itaconic acid, sorbic acid and tartaric acid was observed in the case of 20 kHz treated *L*. *sakei* samples compared to 45 kHz and control. The utilisation of malic acid and galacturonic acid was observed in the case of 130 and 950 kHz samples compared to other ultrasound treated and/or control samples. Among alcohols, higher activity value was observed in all cases for dihydroxyacetone and 2,3-butanedione however, 2-aminoethanol and 2,3-butanedione was utilised only in the case of ultrasound treated samples.

Amino acids as a source of carbon was not utilised for control or ultrasound treatment whereas, alcohols as a source of carbon was utilised by all treated and control samples. Among nitrogen sources, ultrasound treated *L*. *sakei* did not show any utilisation for nitrate, with exception to the control. No utilisation of amino acids as a nitrogen source was also observed for all samples, with the exception of cysteine, tryptophan and ornithine. Some activity (AV = 1) was observed for 20 kHz samples for threonine, valine and citrulline. Amino acids as a nitrogen source was utilised by 20 kHz samples compared to other treatments whereas, peptides as a nitrogen source seems not to be utilised by 45kHz and inorganic form of nitrogen was not utilised by both 20 and 45 kHz. No significant changes in utilisation of organic sulphur was observed among various treatments with some variations observed for 20 kHz samples whereas, in the case of inorganic phosphorus higher activity was observed for the control compared to ultrasound treatment. In the case of phosphorus, organic source of phosphorus was utilised in the case of 20 kHz and control whereas, inorganic source of phosphorus was exclusively utilised by the control compared with ultrasound treated with no utilisation observed for 20 kHz.

**Fig 5:**
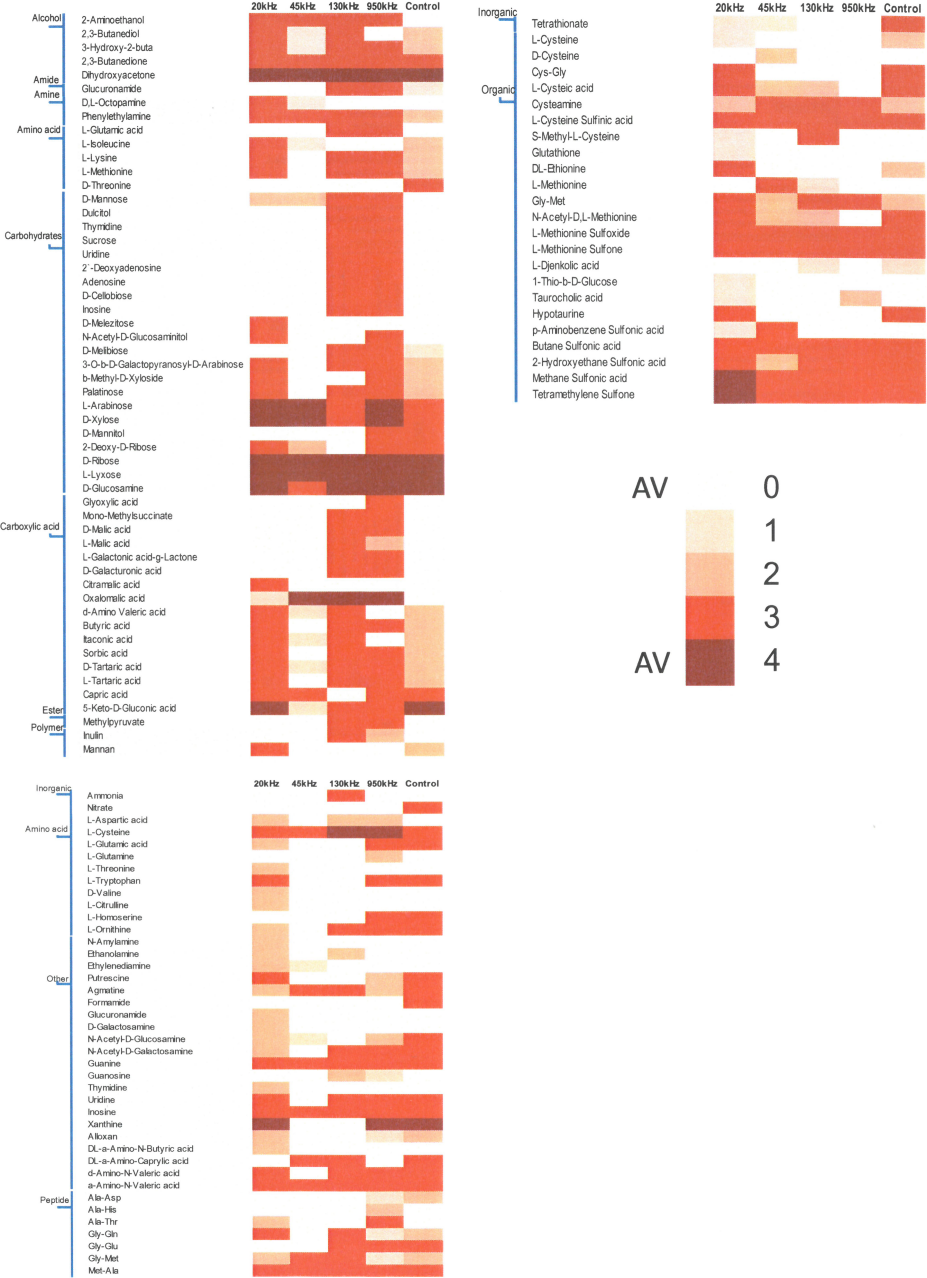
Heatmap of chemicals obtained from phenomic analysis for various PM plates (PM1-2: Carbon source;PM3: Nitrogen source; PM4: Phosphorus and Sulphur source for control and ultrasound treated *L*. *sakei culture*.

**Fig 6:**
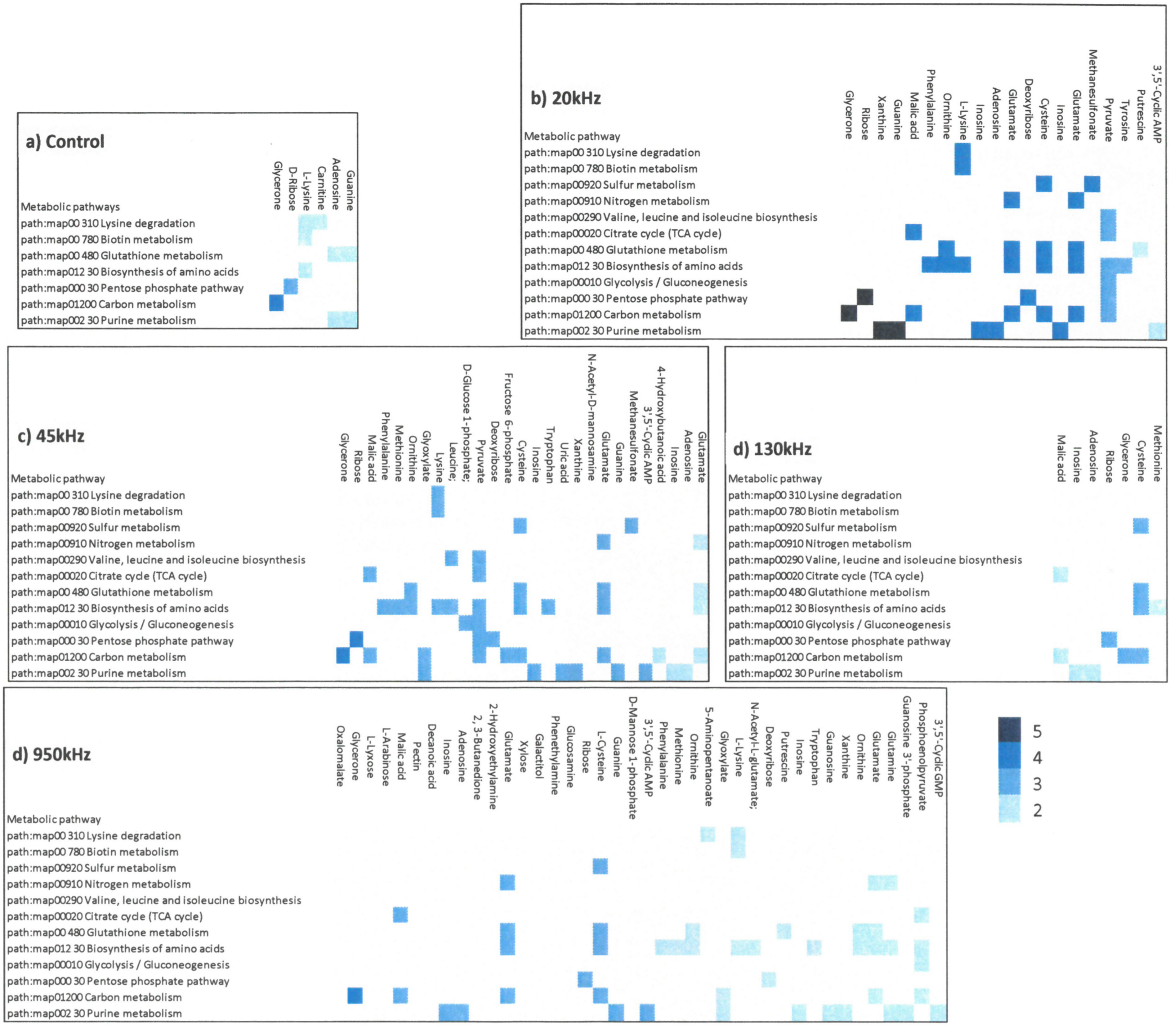
Combined genotypic and phenotypic variability of *L*. *sakei* culture as influenced by various treatments.

The phenotypic variability identified from PM1-4 were combined with genome of the *L*. *sakei* subsp. *carnosus* DSM 15831. Figs 5 and 6 shows that the ultrasound treatment influenced the utilisation of various nutrients resulting in variations in various metabolic pathways. Metabolic reconstruction Citrate cycle (KEGG map20), Glycolysis/ Gluconeogenesis (KEGG map10) and Pentose phosphate pathway (KEGG map 30) were influenced by ultrasound treatment compared to control. Various other metabolic pathways seems influenced by ultrasound treatments were degradation of lysine (KEGG map310), metabolism of biotin (KEGG map780), glutathione (KEGG map480), carbon (KEGG map1200), purine (KEGG map230), biosynthesis of valine, leucine and isoleucine (KEGG map290). Specifically, ultrasound treatment showed highest number of genome related sustrate used differently by ultrasound treated samples compared to control (Fig 5). This difference was noted mainly for biosynthesis of amino acids (KEGG map1230). Further, a higher utilisation of ribose for the ultrasound treated samples compared to the control was observed, ribose sugar is linked to pentose phosphate pathway. A considerable number of nutrients metabolised by ultrasound treated *L*. *sakei* culture associated with the pentose phosphate pathway (KEGG map30) (S2 Fig) were pyruvate (C00022), D-ribose (C00121), D-gluconate (C00257) and deoxyribose (C01801) with varied level of activities; the highest being noted for ribose and deoxyribose in the case of 20 kHz and 45 kHz (AV>5) compared to control.

### Morphological changes

**Fig 7:**
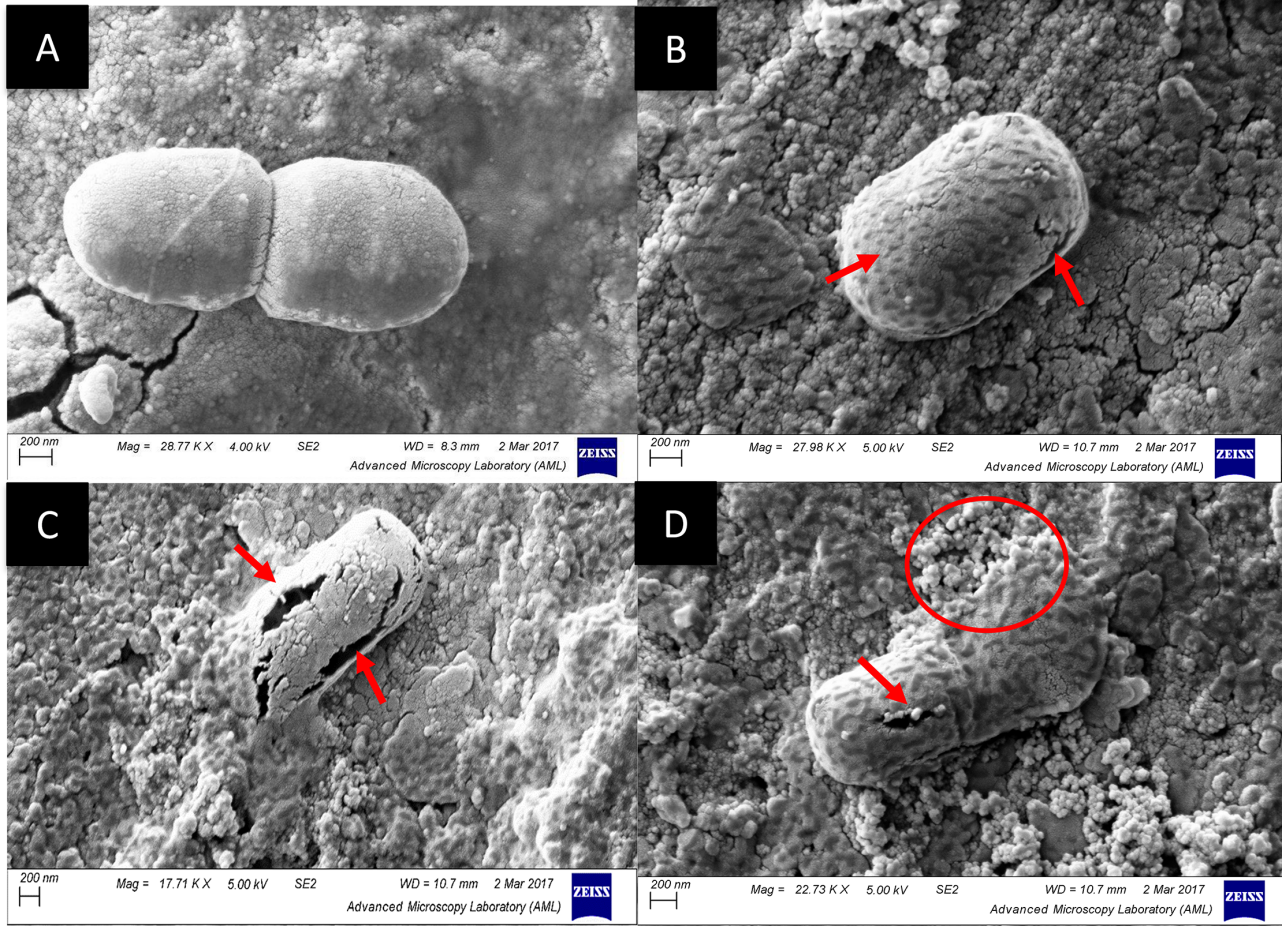
Cryo SEM images of *L*. *sakei* strain (A: Control; B–D: 20 kHz samples). Arrow indicates formation of pores on cellular surface and circle shows the rupture of *L*. *sakei* cells.

Fig 7 shows SEM images of control and 20 kHz treated *L*. *sakei* cells. It shows that the control (A) bacterial cells are intact with uniform surface whereas 20kHz treated *L*. *sakei* cells show formation of pitting (B–D). Some bacterial cells visualised showed physical damage, leading to a formation of pores on the cell surface (highlighted by arrow) which may be reversible, while certain cells within the treated suspension showed rupturing of *L*. *sakei* cells leading to leakage of cellular component (highlighted with a circle, D), causing irreversible damage to the cells.

## Discussion

Previous studies have shown that ultrasound treatment can significantly inactivate bacteria depending on the ultrasound conditions. High power ultrasound treatment has been shown to inactivate various pathogenic and spoilage microorganisms including *Enterobacter aerogenes*, *Bacillus subtilis* and *Staphylococcus epidermidis*, *Escherichia coli* and *Listeria monocytogenes* in various media [28, 29]. Inactivation is mainly due to various physical and chemical phenomena occurring due to inertial and non-inertial cavitation occurring alone or in combination. A previous study by these authors investigated the effect of ultrasound power at a frequency of 20 kHz and showed a significantly higher specific growth rate with a short lag phase at low US power (2.99 W), while a significant decrease (P<0.05) in specific growth rate and increase in lag phase was observed following treatment with increased ultrasound power and treatment time [30].

Use of OD data for the estimation of bacterial growth model parameters has been reported extensively [31]. The growth models investigated in this study are often argued to be of limited biological significance for being empirical in nature compared to mechanistic models. However, empirical models can help in elucidating the effect of a single environmental factor such as temperature, pH, treatment etc. on relevant biological growth parameters i.e. growth rate and lag phase [32]. The Gompertz model has been previously reported to describe the growth behaviour of *Lactobacillus* species in range of media [30, 33–35]. The empirical modelling approach using the Gompertz model approximates cell growth over incubation time and considers the growth inhibition as microbial cells move into the late growth phase[36].

Previous studies have shown the application of various growth models including scale free, Gompertz, logistic, and Richards models to describe the growth behaviour of a range of pathogenic, spoilage and probiotic strain [37–40]. Hence, these models were employed to describe the effect of ultrasound treatment on the growth behaviour of *L*. *sakei*. Previously, a study has shown that a decrease in lag phase is proportional to an increase in initial population because the culture requires less time to reach a concentration which can increase detectable optical density [41]. The longer lag phase for 20 kHz samples can be attributed to the fact it took certain amount of time for injured cells to recover but which then grew at a faster rate compared to other treatments and control. It may be hypothesised that bacteria grew at faster rate due to enhanced movement of nutrients into the cell via pores formed as a result of ultrasound treatment. Studies have shown that the sublethally stressed populations, i.e., the bacteria population which is slightly damaged by environmental conditions are believed to be more susceptible to change and exhibit greater phenotypic variations and have different response to nutrients [42–44] as can be deduced from the variations in metabolic pathways and *L*. *sakei* response to various nutrients investigated in this study. Further, microscopic images (Fig 7) show that ultrasound can induce pits or pores leading to a sublethal or lethal injury to the microbial cells. This injury can be attributed to sonoporation which would have led to the formation of reversible pores which can improve permeability of the cell membrane[2]. However, formation of large size pores may cause permanent cell injury as can be seen from Fig 7D. in a study Wordon, Mortimer [45] observed a extensive boundary damage and disintegration of *S*. *cerevisiae* cells as a result of ultrasound treatment at 20 kHz when exposed to ultrasound.

Studies have shown that low frequency high intensity ultrasound causes inactivation compared to high frequency low intensity ultrasound [46]. Inactivation of various microorganisms in model solution and food matrices has been reported at 20 kHz [47, 48]. The effect of ultrasound on *L*. *sakei* suspensions can be attributed to dispersion effect on *L*. *sakei* clumps, disruption of bacterial cells, modification of cellular activity, either due to introduction of morphological changes on the cell membrane, or cavitation effects within the cell leading to changes in microbial growth parameters and inactivation. Physical and chemical phenomena of ultrasound associated with ultrasound frequencies include agitation, vibration, pressure, shock waves, shear forces, microjets, compression & rarefaction, acoustic streaming, cavitation and formation of free radicals [49]. These effects on the microbial cell are mainly due to physical and chemical mechanisms and/or combination of these occurring due to various types of cavitation namely direct field pressure (Po, kPa), stable cavitation pressure (Ps, kPa) and transient cavitation pressure (Pt, kPa). Po causes oscillation of microbial cells at the fundamental frequency whereas, Ps (non-inertial) cavitation causes oscillation of cavitating bubbles and is responsible for microstreaming. Pt (inertial cavitation) is mainly responsible for microstreaming, formation of shockwaves and jetting due to collapse of cavitating bubble. Collapse of cavitating bubble causes formation of free radicals (e.g. OH^–^) due to sonolysis of water (H_2_O→OH^−^+ H^+^) and formation of hydrogen peroxide.

*L*. *sakei* has an ability to produce all amino acids with exception to aspartic and glutamic acids for its growth and survival [50]. *L*. *sakei* being facultative heterofermentative in nature, hence can utilises glucose for glycolysis and ribose using phosphoketolase pathway. Irrespective of treatments, utilisation of ribose, arabinose, lyxose, xylose was unaffected whereas, no utilisation of glucose was observed. Arabinose utilisation was comparatively higher for 20 kHz, 45 kHz and 950 kHz with AV = 4 compared to 130 kHz and control with AV = 3. *L*. *sakei* are able to ferment many pentoses including ribose, arabinose, ribulose and xylulose by using the phosphoketolase pathway[51]. Phenotypic characteristics of various *L*. *sakei* strains including subsp. *carnosus* studied by Koort, Vandamme [25] showed arabinose, galactose, maltose, melibiose sucrose, trehalose can be utilised with weakly positive results for β-Gentiobiose and Gluconate. The genome of *L*. *sakei* strain was mapped with phenotypic variations for ultrasound treated and control *L*. *sakei* culture. Linking genome with high throughput phenotypic characteristics revealed significant differences in metabolic responses to various ultrasonic frequencies.

The genetic diversity of *L*. *sakei* strains has been studied and reported extensively [14, 52, 53]. Previous studies have shown that phenotypic variations exist and the ability of strains to utilise carbohydrates varies significantly depending on the environment and growing conditions [54]. Various metabolic pathways were influenced as a result of the ultrasound treatment and concomitant nutrient utilisation. The focus of the following discussion is restricted to the utilisation of ribose sugar and the effect of ultrasound treatment on the phosphate pentose pathway. The *L*. *sakei* strain employed in this study has been reported to have limited utilisation of ribose [14]. The major heterolactic fermentation pathway of ribose is via the phosphoketolase pathway. In accordance with the literature, low activity was observed for ribose utilisation in the case of the control whereas, higher activity was observed in the case of ultrasound treated *L*. *sakei* culture, indicating possible changes in protein expression for ribose utilisation via phosphate pentose pathway. The utilisation of ribose sugar leading to an impaired growth on ribose is reported due to the inactivation of *rbsK* which encodes ribokinase. Moreover, both *rbsK* and *rbsR*, encoding a ribokinase are shown to be divergent in various strains of *L*. *sakei* [22] which may have been expressed differently owing to ultrasound treatment. Catabolic pathways for utilisation of various sugars exist in almost all strains identified to date for *L*. *sakei* species. Release of ribose via ATP hydrolysis with intermediates such as inosine and IMP are the most abundant intermediates of ATP breakdown. Metabolism of ribose for energy production in *L*. *sakei* include conversion of Ribose-1P→Ribose-5P (enzyme:phosphopentomutase); Ribose→Ribose-5P (Enzyme: Ribokinases) and conversion of Ribose-5P →Ribulose-5P (enzyme: ribose-5-phosphate isomerase)[55]. Metabolism of pyruvate is very important for the phosphoketolase pathaway which is converted to lactate by a NAD-dependent lactate dehydrogenase. The phosphoketolase pathway along with glycolysis is responsible for production of lactic acid depending on the metabolism of glucose or ribose.

The comparative PM analysis of the growth or no-growth response of ultrasound treated or control strains provided significant differences as shown in Figs 5 and 6. Higher metabolic activity of ultrasound treated culture may be due to a change in gene expression leading to the utilisation of various nutrients.

Phenotypic alterations and application of genomic-phenomic approach of a culture subjected to environmental stress e.g. adaption to cold environment has been successfully demonstrated [56]. Phenotypic variations identified as a result of ultrasound treatment and links associated with the various metabolic pathways demonstrates the relevance of PM technology in providing in-depth analysis of *L*. *sakei* growth which may be useful in understanding the influence of processing technologies on microorganisms, which to now has been restricted mainly for inactivation and growth studies. This study signifies that the genomic-phenomic approach for understanding the key underlying mechanism and adaptive response to emerging technologies. Understanding phenotypic variations and differences in nutrient utilisation will also enable tailoring the food processes for specialised applications of probiotic strains.

## Supporting information

S1 Fig Genome statistics of *L*. *sakei* strain employed showing the size of proteome, and various metabolic reaction investigated using DuctApe analysis. (PDF)

S2 Fig Pentose phosphate metabolic pathway analysis using the DuctApe (KEGG map0030). Boxes with EC number represents reaction catalysed by enzymes whereas small circles represents compounds. Compounds circled showed higher metabolic activity for ultrasound treated culture compared to control. These compounds are mediated by ribokinase. [EC:2.7.1.15].
(PDF)

## References

[1] Karshafian R, Samac S, Bevan PD, Burns PN. Microbubble mediated sonoporation of cells in suspension: Clonogenic viability and influence of molecular size on uptake. Ultrasonics. 2010;50(7):691–7. doi: 10.1016/j.ultras.2010.01.009 20153497 10.1016/j.ultras.2010.01.009

[2] Lentacker I, De Cock I, Deckers R, De Smedt S, Moonen C. Understanding ultrasound induced sonoporation: definitions and underlying mechanisms. Advanced drug delivery reviews. 2014;72:49–64. doi: 10.1016/j.addr.2013.11.008 24270006 10.1016/j.addr.2013.11.008

[3] Yeo S-K, Liong M-T. Effects and applications of sub-lethal ultrasound, electroporation and UV radiations in bioprocessing. Annals of Microbiology. 2013;63(3):813–24.

[4] Yang F, Gu N, Chen D, Xi X, Zhang D, Li Y, et al Experimental study on cell self-sealing during sonoporation. Journal of Controlled Release. 2008;131(3):205–10. doi: 10.1016/j.jconrel.2008.07.038 18727944 10.1016/j.jconrel.2008.07.038

[5] Moncada M, Aryana KJ, Boeneke C. Effect of Mild Sonication Conditions on the Attributes of Lactobacillus delbrueckii ssp. bulgaricus LB-12. 2012.

[6] Monsen T, Lövgren E, Widerström M, Wallinder L. In Vitro Effect of Ultrasound on Bacteria and Suggested Protocol for Sonication and Diagnosis of Prosthetic Infections. Journal of Clinical Microbiology. 2009;47(8):2496–501. doi: 10.1128/JCM.02316-08 19535525 10.1128/JCM.02316-08 PMC2725697

[7] Lanchun S, Bochu W, Liancai Z, Jie L, Yanhong Y, Chuanren D. The influence of low-intensity ultrasonic on some physiological characteristics of Saccharomyces cerevisiae. Colloids and Surfaces B: Biointerfaces. 2003;30(1):61–6.

[8] Lanchun S, Bochu W, Zhiming L, Chuanren D, Chuanyun D, Sakanishi A. The research into the influence of low-intensity ultrasonic on the growth of S. cerevisiaes. Colloids and Surfaces B: Biointerfaces. 2003;30(1):43–9.

[9] Jomdecha C, Prateepasen A. Effects of pulse ultrasonic irradiation on the lag phase of Saccharomyces cerevisiae growth. Letters in applied microbiology. 2011;52(1):62–9. doi: 10.1111/j.1472-765X.2010.02966.x 21143488 10.1111/j.1472-765X.2010.02966.x

[10] Nguyen TMP, Lee YK, Zhou W. Stimulating fermentative activities of bifidobacteria in milk by highintensity ultrasound. International Dairy Journal. 2009;19(6–7):410–6.

[11] Nguyen TMP, Lee YK, Zhou W. Effect of high intensity ultrasound on carbohydrate metabolism of bifidobacteria in milk fermentation. Food Chemistry. 2012;130(4):866–74.

[12] Yang S, Zhang H, Wang W. The ultrasonic effect on the mechanism of cholesterol oxidase production by Brevibacterium sp. African Journal of Biotechnology. 2010;9(17):2574–8.

[13] Castellano PH, Holzapfel WH, Vignolo GM. The control of Listeria innocua and Lactobacillus sakei in broth and meat slurry with the bacteriocinogenic strain Lactobacillus casei CRL705. Food Microbiology. 2004;21(3):291–8.

[14] McLeod A, Nyquist OL, Snipen L, Naterstad K, Axelsson L. Diversity of Lactobacillus sakei strains investigated by phenotypic and genotypic methods. Systematic and Applied Microbiology. 2008;31(5):393–403. doi: 10.1016/j.syapm.2008.06.002 18678454 10.1016/j.syapm.2008.06.002

[15] Tremaroli V, Bäckhed F. Functional interactions between the gut microbiota and host metabolism. Nature. 2012;489(7415):242–9. doi: 10.1038/nature11552 22972297 10.1038/nature11552

[16] Holmes E, Li JV, Athanasiou T, Ashrafian H, Nicholson JK. Understanding the role of gut microbiome–host metabolic signal disruption in health and disease. Trends in microbiology. 2011;19(7):349–59. doi: 10.1016/j.tim.2011.05.006 21684749 10.1016/j.tim.2011.05.006

[17] Greetham D. Phenotype microarray technology and its application in industrial biotechnology. Biotechnology letters. 2014;36(6):1153–60. doi: 10.1007/s10529-014-1481-x 24563312 10.1007/s10529-014-1481-x

[18] Tiwari B, Muthukumarappan K, O’Donnell C, Cullen P. Effects of sonication on the kinetics of orange juice quality parameters. Journal of Agricultural and Food Chemistry. 2008;56(7):2423–8. doi: 10.1021/jf073503y 18321054 10.1021/jf073503y

[19] Galardini M, Mengoni A, Biondi EG, Semeraro R, Florio A, Bazzicalupo M, et al DuctApe: A suite for the analysis and correlation of genomic and OmniLog™ Phenotype Microarray data. Genomics. 2014;103(1):1–10. doi: 10.1016/j.ygeno.2013.11.005 24316132 10.1016/j.ygeno.2013.11.005

[20] Kanehisa M, Goto S. KEGG: kyoto encyclopedia of genes and genomes. Nucleic acids research. 2000;28(1):27–30. 10592173 10.1093/nar/28.1.27 PMC102409

[21] NCBI. Availabe onlie at https://www.ncbi.nlm.nih.gov/genome/1664?genome_assembly_id=256489 (Access date: 03 June 2017). 2017.

[22] Nyquist OL, McLeod A, Brede DA, Snipen L, Aakra Å, Nes IF. Comparative genomics of Lactobacillus sakei with emphasis on strains from meat. Molecular Genetics and Genomics. 2011;285(4):297–311. doi: 10.1007/s00438-011-0608-1 21369871 10.1007/s00438-011-0608-1

[23] Chaillou S, Daty M, Baraige F, Dudez A-M, Anglade P, Jones R, et al Intraspecies genomic diversity and natural population structure of the meat-borne lactic acid bacterium Lactobacillus sakei. Applied and environmental microbiology. 2009;75(4):970–80. doi: 10.1128/AEM.01721-08 19114527 10.1128/AEM.01721-08 PMC2643555

[24] Chaillou S, Lucquin I, Najjari A, Zagorec M, Champomier-Vergès M-C. Population genetics of Lactobacillus sakei reveals three lineages with distinct evolutionary histories. PLoS One. 2013;8(9):e73253 doi: 10.1371/journal.pone.0073253 24069179 10.1371/journal.pone.0073253 PMC3777942

[25] Koort J, Vandamme P, Schillinger U, Holzapfel W, Björkroth J. Lactobacillus curvatus subsp. melibiosus is a later synonym of Lactobacillus sakei subsp. carnosus. International journal of systematic and evolutionary microbiology. 2004;54(5):1621–6. 15388719 10.1099/ijs.0.63164-0

[26] Baranyi J, Roberts TA. A dynamic approach to predicting bacterial growth in food. International journal of food microbiology. 1994;23(3–4):277–94. 7873331 10.1016/0168-1605(94)90157-0

[27] Baranyi J, Roberts TA, Mcclure P. Some properties of a nonautonomous deterministic growth model describing the adjustment of the bacterial population to a new environment. Mathematical Medicine and Biology. 1993;10(4):293–9.

[28] Gao S, Hemar Y, Ashokkumar M, Paturel S, Lewis GD. Inactivation of bacteria and yeast using high-frequency ultrasound treatment. Water Research. 2014;60:93–104. doi: 10.1016/j.watres.2014.04.038 24835956 10.1016/j.watres.2014.04.038

[29] Gera N, Doores S. Kinetics and mechanism of bacterial inactivation by ultrasound waves and sonoprotective effect of milk components. Journal of food science. 2011;76(2):M111–M9. doi: 10.1111/j.1750-3841.2010.02007.x 21535773 10.1111/j.1750-3841.2010.02007.x

[30] Ojha KS, Kerry JP, Alvarez C, Walsh D, Tiwari BK. Effect of high intensity ultrasound on the fermentation profile of Lactobacillus sakei in a meat model system. Ultrasonics sonochemistry. 2016;31:539–45. doi: 10.1016/j.ultsonch.2016.01.001 26964981 10.1016/j.ultsonch.2016.01.001

[31] Dalgaard P, Ross T, Kamperman L, Neumeyer K, McMeekin TA. Estimation of bacterial growth rates from turbidimetric and viable count data. International Journal of Food Microbiology. 1994;23(3):391–404. 7873339 10.1016/0168-1605(94)90165-1

[32] Leroy F, De Vuyst L. Growth of the bacteriocin-producingLactobacillus sakei strain CTC 494 in MRS broth is strongly reduced due to nutrient exhaustion: a nutrient depletion model for the growth of lactic acid bacteria. Applied and Environmental Microbiology. 2001;67(10):4407–13. doi: 10.1128/AEM.67.10.4407-4413.2001 11571136 10.1128/AEM.67.10.4407-4413.2001 PMC93183

[33] Aryani D, Zwietering M, den Besten H. The effect of different matrices on the growth kinetics and heat resistance of Listeria monocytogenes and Lactobacillus plantarum. International Journal of Food Microbiology. 2016;238:326–37. doi: 10.1016/j.ijfoodmicro.2016.09.012 27723494 10.1016/j.ijfoodmicro.2016.09.012

[34] Siragusa S, De Angelis M, Calasso M, Campanella D, Minervini F, Di Cagno R, et al Fermentation and proteome profiles of Lactobacillus plantarum strains during growth under food-like conditions. Journal of proteomics. 2014;96:366–80. doi: 10.1016/j.jprot.2013.11.003 24231110 10.1016/j.jprot.2013.11.003

[35] Mechmeche M, Kachouri F, Yaghlane HB, Ksontini H, Setti K, Hamdi M. Kinetic analysis and mathematical modeling of growth parameters of Lactobacillus plantarum in protein-rich isolates from tomato seed. Food Science and Technology International. 2017;23(2):128–41. doi: 10.1177/1082013216665706 27574029 10.1177/1082013216665706

[36] Zwietering M, Jongenburger I, Rombouts F, Van't Riet K. Modeling of the bacterial growth curve. Applied and Environmental Microbiology. 1990;56(6):1875–81. 16348228 10.1128/aem.56.6.1875-1881.1990 PMC184525

[37] Sant’Ana AS, Franco BDGM, Schaffner DW. Modeling the growth rate and lag time of different strains of Salmonella enterica and Listeria monocytogenes in ready-to-eat lettuce. Food Microbiology. 2012;30(1):267–73. doi: 10.1016/j.fm.2011.11.003 22265311 10.1016/j.fm.2011.11.003

[38] Wijtzes T, Rombouts F, Kant-Muermans M, Van’t Riet K, Zwietering M. Development and validation of a combined temperature, water activity, pH model for bacterial growth rate of Lactobacillus curvatus. International journal of food microbiology. 2001;63(1):57–64. 11205954 10.1016/s0168-1605(00)00401-3

[39] Tomás MSJ, Labanda EBd, de Ruiz Holgado AP, Nader-Macías ME. Estimation of vaginal probiotic lactobacilli growth parameters with the application of the Gompertz model. Canadian journal of microbiology. 2002;48(1):82–92. 11888167 10.1139/w01-135

[40] Sharma V, Mishra HN. Unstructured kinetic modeling of growth and lactic acid production by Lactobacillus plantarum NCDC 414 during fermentation of vegetable juices. LWT-Food Science and Technology. 2014;59(2):1123–8.

[41] Pla M-L, Oltra S, Esteban M-D, Andreu S, Palop A. Comparison of primary models to predict microbial growth by the plate count and absorbance methods. BioMed research international. 2015;2015. 10.1155/2015/365025 PMC4619785 26539483

[42] Mossel D, Van Netten P, editors. Harmful effects of selective media on stressed micro-organisms: nature and remedies. Society for Applied Bacteriology symposium series; 1984. 6387937

[43] Flowers RS, Ordal ZJ. Current methods to detect stressed staphylococci. Journal of Food Protection. 1979;42(4):362–7. 10.4315/0362-028X-42.4.362 30812193

[44] Roszak D, Colwell R. Survival strategies of bacteria in the natural environment. Microbiological reviews. 1987;51(3):365 3312987 10.1128/mr.51.3.365-379.1987 PMC373117

[45] Wordon BA, Mortimer B, McMaster LD. Comparative real-time analysis of Saccharomyces cerevisiae cell viability, injury and death induced by ultrasound (20 kHz) and heat for the application of hurdle technology. Food Research International. 2012;47(2):134–9.

[46] Radel S, McLoughlin AJ, Gherardini L, Doblhoff-Dier O, Benes E. Viability of yeast cells in well controlled propagating and standing ultrasonic plane waves. Ultrasonics. 2000;38(1–8):633–7. 10829741 10.1016/s0041-624x(99)00211-5

[47] Shamila-Syuhada AK, Chuah L-O, Wan-Nadiah WA, Cheng LH, Alkarkhi AF, Effarizah ME, et al Inactivation of microbiota and selected spoilage and pathogenic bacteria in milk by combinations of ultrasound, hydrogen peroxide, and active lactoperoxidase system. International Dairy Journal. 2016;61:120–5.

[48] Barba FJ, Koubaa M, do Prado-Silva L, Orlien V, de Souza Sant’Ana A. Mild processing applied to the inactivation of the main foodborne bacterial pathogens: A review. Trends in Food Science & Technology. 2017.

[49] Ojha KS, Mason TJ, O’Donnell CP, Kerry JP, Tiwari BK. Ultrasound technology for food fermentation applications. Ultrasonics sonochemistry. 2017;34:410–7. doi: 10.1016/j.ultsonch.2016.06.001 27773263 10.1016/j.ultsonch.2016.06.001

[50] Champomier-Vergès M-C, Chaillou S, Cornet M, Zagorec M. Lactobacillus sakei: recent developments and future prospects. Research in Microbiology. 2001;152(10):839–48. 11766959 10.1016/s0923-2508(01)01267-0

[51] McLeod A, Zagorec M, Champomier-Vergès M-C, Naterstad K, Axelsson L. Primary metabolism in Lactobacillus sakei food isolates by proteomic analysis. BMC microbiology. 2010;10(1):120. 20412581 10.1186/1471-2180-10-120 PMC2873491

[52] Jensen H, Grimmer S, Naterstad K, Axelsson L. In vitro testing of commercial and potential probiotic lactic acid bacteria. International journal of food microbiology. 2012;153(1):216–22. 22177712 10.1016/j.ijfoodmicro.2011.11.020

[53] Claesson MJ, Van Sinderen D, O'Toole PW. The genus Lactobacillus—a genomic basis for understanding its diversity. FEMS microbiology letters. 2007;269(1):22–8. doi: 10.1111/j.1574-6968.2006.00596.x 17343688 10.1111/j.1574-6968.2006.00596.x

[54] Chiaramonte F, Anglade P, Baraige F, Gratadoux J-J, Langella P, Champomier-Vergès M-C, et al Analysis of Lactobacillus sakei mutants selected after adaptation to the gastrointestinal tracts of axenic mice. Applied and environmental microbiology. 2010;76(9):2932–9. doi: 10.1128/AEM.02451-09 20208026 10.1128/AEM.02451-09 PMC2863443

[55] Chaillou S, Champomier-Vergès M-C, Cornet M, Crutz-Le Coq A-M, Dudez A-M, Martin V, et al The complete genome sequence of the meat-borne lactic acid bacterium Lactobacillus sakei 23K. Nature biotechnology. 2005;23(12):1527–33. doi: 10.1038/nbt1160 16273110 10.1038/nbt1160

[56] Mocali S, Chiellini C, Fabiani A, Decuzzi S, de Pascale D, Parrilli E, et al Ecology of cold environments: new insights of bacterial metabolic adaptation through an integrated genomic-phenomic approach. Scientific Reports. 2017;7. 10.1038/s41598-017-00876-4 PMC5429795 28404986

