# Supplementary figures and images for: Integrated phenotypic-genotypic approach to understand the influence of ultrasound on metabolic response of Lactobacillus sakei
Source: PLoS One. 2018 Jan 25;13(1):e0191053. doi: 10.1371/journal.pone.0191053 (PMC5784923; doi:10.1371/journal.pone.0191053)

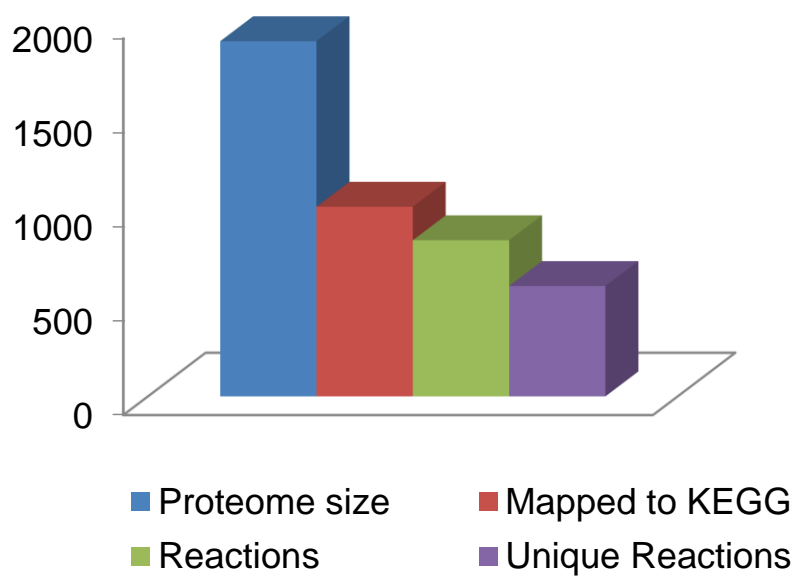

Supplement: S1 Fig — (PDF) [file pone.0191053.s001.pdf]

# PENTOSE PHOSPHATE PATHWAY

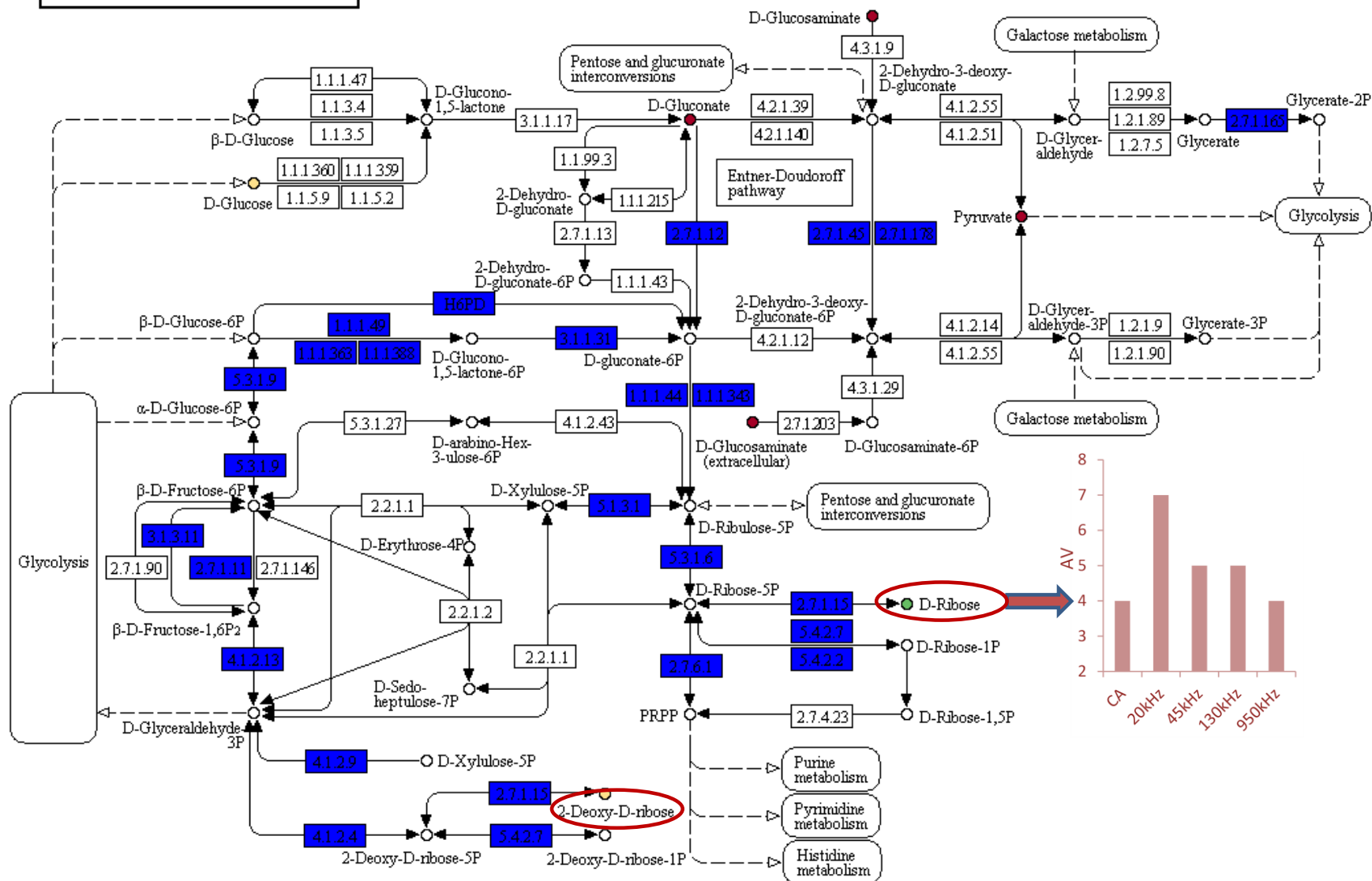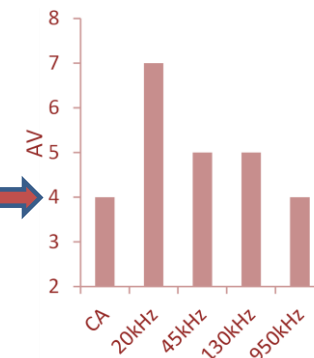

Supplement: S2 Fig — Boxes with EC number represents reaction catalysed by enzymes whereas small circles represents compounds. Compounds circled showed higher metabolic activity for ultrasound treated culture compared to control. These compounds are mediated by ribokinase. [EC:2.7.1.15]. (PDF) [file pone.0191053.s002.pdf]
